# Anti-*Toxoplasma* Activities of Some Egyptian Plant Extracts: An In Vitro Study

**DOI:** 10.1007/s11686-022-00633-2

**Published:** 2022-10-30

**Authors:** Sara T. Elazab, Fadwa M. Arafa

**Affiliations:** 1 Department of Pharmacology, Faculty of Veterinary Medicine, Mansoura University, Mansoura, 35516 Egypt; 2 Department of Medical Parasitology, Faculty of Medicine, Alexandria University, Alexandria, Egypt

**Keywords:** *Toxoplasma gondii*, Selectivity index, Cytotoxicity, Plant extracts, *Raphanus sativus*

## Abstract

**Purpose:**

Toxoplasmosis is a globally widespread parasitic disease which causes major health problems in human and animals. This research was conducted to assess the effect of some Egyptian herbal extracts against *Toxoplasma gondii* (*T. gondii)* tachyzoites in vitro.

**Methods:**

The methanol extracts of *Withania somnifera, Cyper rotundus*, *Acacia nilotica***,** *Chrysanthemum cinerariae folium*, *Anethum graveolens*, *Raphanus sativus*, *Ceratonia siliqua*, *Elettaria cardamomum* and *Cuminum cyminum* were tested against *T. gondii* tachyzoites.

**Results:**

Among the tested plants, the extracts from *Raphanus sativus, Cuminum cyminum,* and *Ceratonia siliqua* exhibited high *anti-Toxoplasma* activities at 50 µg/ml, relative to sulfadiazine. They showed low IC_50_ values on *T. gondii* (7.92, 9.47 and 13.52 µg/ml, respectively) and high selectivity index values (100.79, 59.19, and 29.05, respectively). Scanning electron microscopy (SEM) findings indicated evident morphological changes in tachyzoites treated with these three herbal extracts.

**Conclusion:**

*Raphanus sativus*, *Ceratonia siliqua*, and *Cuminum cyminum* methanol extracts could be promising sources of new medicament for toxoplasmosis.

*Toxoplasma gondii* (*T. gondii)* is an intracellular apicomplexan protozoan parasite which causes a widely distributed zoonotic disease, toxoplasmosis, in warm-blooded animals [1]. Almost 30% of the world’s population is affected by toxoplasmosis [2]. The severity of toxoplasmosis differs according to the host immune status [3]. During the gestation period, *T. gondii* can cross the placental barrier and infect the fetus causing fetal malformations or abortion [4]. Nowadays, the combination of sulfonamide drugs and pyrimethamine is regarded as the first-line therapy for toxoplasmosis [5]. These drugs elicit their effects by suppressing the synthesis of DNA and or RNA of *T. gondii* via inhibiting *Toxoplasma* folic acid metabolism [6]. Nevertheless, these medicines have limitations due to numerous undesirable side effects such as hypersensitivity reactions, bone marrow suppression, and hematological disorders, as well as their restricted efficacy in eradicating tissue cysts [7]. Thus, the development of new, efficient, and more tolerable therapies is imperative.

Medicinal herbal extracts are promising sources of novel remedies, owing to their high content of diverse bioactive compounds [8]. Previously, numerous drugs for eradicating parasitic infestations have been derived from plants, for instance, artemisinin and quinine as a therapy for malaria [9]. The goal of the present study was to investigate the potential anti-*Toxoplasma* effects of methanol extracts of some Egyptian plants. The plants utilized in this research, such as *Withania somnifera* [10, 11], *Cyper rotundus* [12], *Acacia nilotica* [13], *Chrysanthemum cinerariae folium* [14], *Anethum graveolens* [15], *Raphanus sativus* [16], *Ceratonia siliqua* [17], *Elettaria cardamomum* [18], and *Cuminum cyminum* [19], have been previously recorded to possess antiprotozoal and anthelmintic. However, to the author’s knowledge, no reports are available regarding their possible effects against *T. gondii*.

Samples of *Withania somnifera* (Ashwagandha), *Cyper rotundus* (purple nutsedge), *Acacia nilotica* (gum arabic tree**),** *Chrysanthemum cinerariae folium* (pyrethrum), *Anethum graveolens* (dill), *Raphanus sativus* (radish), *Ceratonia siliqua* (carob), *Elettaria cardamomum* (true cardmom), and *Cuminum cyminum* (cumin) were brought from herbal drug store in Mansoura, Egypt. The conventional uses of the selected plants are listed in Table 1. Dried plants were crushed into tiny pieces and were extracted employing 70% methanol for 48 h. The resulting crude extracts were prepared at 100 mg/ml in dimethylsulfoxide (DMSO) and stored at − 30 °C until use.

**Table 1:** Activities of methanolic extracts from some plants on *Toxoplasma gondii* at concentrations 50 µg/ml and 10 µg/ml

Plants	Plant part	Traditional uses	References	% Inhibition of *T. gondii* RH at concentrations (µg/ml)	
				50	10
*Withania somnifera* (Ashwagandha)	Leaf	Used as aphrodisiac, for treatment of impotency, nervous exhaustion, insomnia, chronic fatigue, skin problems and coughing	[20]	40.99 ± 6.54	24.52 ± 7.48
*Cyper rotundus *(Purple nutsedge)	Leaf	For treating gastrointestinal disorders, menstrual disturbance, bronchitis, leprosy, epilepsy and urolithiasis	[21]	17.24 ± 4.59	3.83 ± 1.76
*Acacia nilotica *(Gum arabic tree**)**	Leaf	As a therapy for diabetes mellitus, bronchitis, tuberculosis and pharyngitis	[22]	43.29 ± 10.68	27.20 ± 5.78
*Chrysanthemum cinerariae folium* (Pyrethrum)	Flower	To cure chest pain, hypertension, allergy, sore throat and headache	[23]	9.96 ± 2.89	4.98 ± 4.03
*Anethum graveolens* (Dill)	Seed	As a diuretic, stomachic and to cure gastrointestinal upset	[24]	31.03 ± 12.00	11.49 ± 5.26
*Raphanus sativus *(Radish)	Seed	For treating dysentery, constipation, hypertension and chronic tracheitis	[25]	73.56 ± 4.14	46.74 ± 8.62
*Ceratonia silique *(Carob)	Fruit	Used as antidiabetic, antidiarrheal, diuretic and antiatherosclerotic	[26]	69.35 ± 9.29	35.63 ± 4.59
*Elettaria cardamomum* (True cardmom)	Seed	As a treatment for cardiac disorders, constipation, urinary problems and tuberculosis	[27]	59.38 ± 12.66	28.73 ± 8.67
*Cuminum cyminum* (Cumin)	Seed	Used as anthelmintic agent, stomachic, carminative, antipyretic, diuretic, tonic and antidiabetic	[28]	77.78 ± 6.73	40.23 ± 13.55

Reference control (1 mg/ml sulfadiazine) exhibited anti*-Toxoplasma* activities with % of RH growth suppression of 61.30 ± 14.37. Data are presented as mean ± SD (*n* = 3)

The potential cytotoxic effect of these herbal extracts was assessed on Vero cells (African green monkey cells supplied from the National Cancer Institute, Cairo, Egypt) based on the technique of Montazeri et al. [29]. In brief, Vero cells were seeded in 96-well plate with 180 µl Roswell Park Memorial Institute medium (RPMI 1640 medium, Sigma, St. Louis, MO, USA) in every well (density, 2 × 10^4^ cells/ well in RPMI 1640 medium containing 10% FBS, and 100 U/ml penicillin, 100 μg/ml streptomycin) and incubated at 37 °C under 5% CO_2_ for 24 h. After that, the cells were subjected to the tested extracts at concentrations of 15.625, 31.25, 62.5, 125, 250, 500 and 1000 μg/ml. Following 24 h incubation, MTT solution (3-[4, 5-dimethylthiazol-2-yl]-2, 5-diphenyltetrazolium bromide) was added to the cultures to measure cell viability. The optical density was determined at 490 nm with a microplate reader (Benchmark, Bio-Rad, USA). The growth inhibition (%) was calculated. The half maximal inhibitory concentration (IC_50_) value of every plant extract on Vero cells was evaluated using GraphPad prism 5 software (GraphPad Software Inc., La Jolla, CA, USA).

To test the effect of herbal extracts on intracellular *T. gondii* invitro, Vero cells were plated in 96-well plate (density 2 × 10^4^ cells/well/180 μl RPMI 1640 medium supplemented with10% FBS) and kept to grow at 37 °C in 5% CO_2_. After 24 h of incubation, *T. gondii* RH tachyzoites (obtained from Medical Parasitology Department, Faculty of Medicine, Alexandria University, Egypt) were added to the wells (parasite: cell ratio = 5:1). 24 h later, the culture medium was changed and the infected cells were exposed to the plant extracts dissolved in RPMI 1640 medium at 10 and 50 µg/ml. Wells with RPMI 1640 medium or sulfadiazine only were considered as negative and positive controls, respectively. Thereafter, MTT solution was added and incubated for 4 h. The optical absorbance was detected at 490 nm. The percentage of *T. gondii* tachyzoites growth inhibition (GI) was estimated based on the following equation:

$${\text{GI}} = \frac{{{\text{At}} - {\text{Ac}}}}{{{\text{Ac}}}} \times \;100,$$

in which At and Ac reflect the absorbance of the treated cells and control, respectively.

The tested extracts exhibiting percentage of inhibition of tachyzoites growth at 50 µg/ml more than that obtained by sulfadiazine were further analyzed at concentrations (0.78–100 µg /ml) to evaluate their IC_50_ values against *T. gondii.* GraphPad prism 5 software was employed to estimate the IC_50_ values of the extracts on *T. gondii*. Selectivity index (SI) of the samples was calculated using the following equation as described by Montazeri et al. [29]:

$${\text{SI}} = \frac{{{\text{IC}}50{\text{ of the herbal extracts on Vero cells}}}}{{{\text{IC}}50{\text{ of the herbal extracts on }}T. gondii}}$$

To assess the anti-*Toxoplasma* activities of the methanol extracts of *Raphanus sativus*, *Ceratonia siliqua, and Cuminum cyminum *by scanning electron microscopy (SEM), following incubation of 10^5^ *Toxoplasma* tachyzoites with each plant extract (at 10 µg/ml) or sulfadiazine (at 100 µg/ml) for 2 h, tachyzoites were loaded on a slide, fixed with 2% paraformaldehyde and 2.5% glutaraldehyde in sodium cacodylate buffer (0.1 M, pH 7.4) and rinsed with cacodylate buffer. Thereafter, the slide was exposed to 1% osmium tetroxide buffer for 2–4 h. Then, they were dehydrated in serial ascending concentrations of ethanol. Finally, the tachyzoites were embedded in Epon Resin, and 20 nm gold particles were utilized for coating prior to investigation using a JOEL-JSM-IT 200, Japan, scanning microscope [30].

The effects of the herbal extracts on *T. gondii* at concentrations 50 and 10 µg/ml are elucidated in Table 1. They showed varying levels of anti*-Toxoplamal* effects at 50 µg/ml, starting from 9.96 up to 77.78% RH growth inhibition. From the nine examined plants, the extracts derived from *Raphanus sativus*, *Cuminum cyminum*, and *Ceratonia siliqua* displayed higher % of RH growth inhibition relative to sulfadiazine at 1 mg/ml (61.30%). Moreover, as presented in Table 2, these three herbal extracts revealed lower IC50 values (7.92, 9.47, and 13.52 µg/ml, respectively) than sulfadiazine (94.41 µg/ml). In addition, based on the “hit” criteria (SI ≥ 10) previously reported by Banzragchgarav et al. [31], the three plants exhibited promising SI values of 100.79, 59.19 and 29.05, respectively. The SI value was regarded as a reliable marker for the selective activity of the remedy [32].

**Table 2:** Half maximal inhibitory concentration (IC50) of methanol herbal extracts displaying anti-*Toxoplasma* activity and their associated selectivity indexes

Herbal extract	IC_50_ (µg/m*l*)		Selectivity index (SI)
	Vero cells	*T. gondii* RH	
*Raphanus sativus* (radish)	798.3 ± 48.10	7.92 ± 1.14	100.79
*Ceratonia siliqua* (carob)	392.8 ± 66.44	13.52 ± 4.85	29.05
*Cuminum cyminum* (cumin)	560.6 ± 51.91	9.47 ± 3.98	59.19
Sulfadiazine	> 1,000	94.41 ± 4.44	ND

IC_50_ values were estimated depending on three independent experiments. Data are presented as mean ± SD

*ND* not detected

Furthermore, morphological analysis of untreated *T. gondii* tachyzoites using SEM exhibited crescent shape with smooth surface (Fig. 1A). In contrast, tachyzoites treated with *Raphanus sativus *methanol extract showed numerous protrusions (arrow), ridges and depressions (Fig. 1C, D). Moreover, those exposed to *Ceratonia siliqua *extract revealed loss of crescentic morphology and ballooning with multiple crystalline deposits on the surface (Fig. 1E, F). Meanwhile, tachyzoites treated with *Cuminum cyminum *extract displayed irregular rough surface with multiple furrows and deep depressions (Fig. 1G, H).

**Fig. 1:**
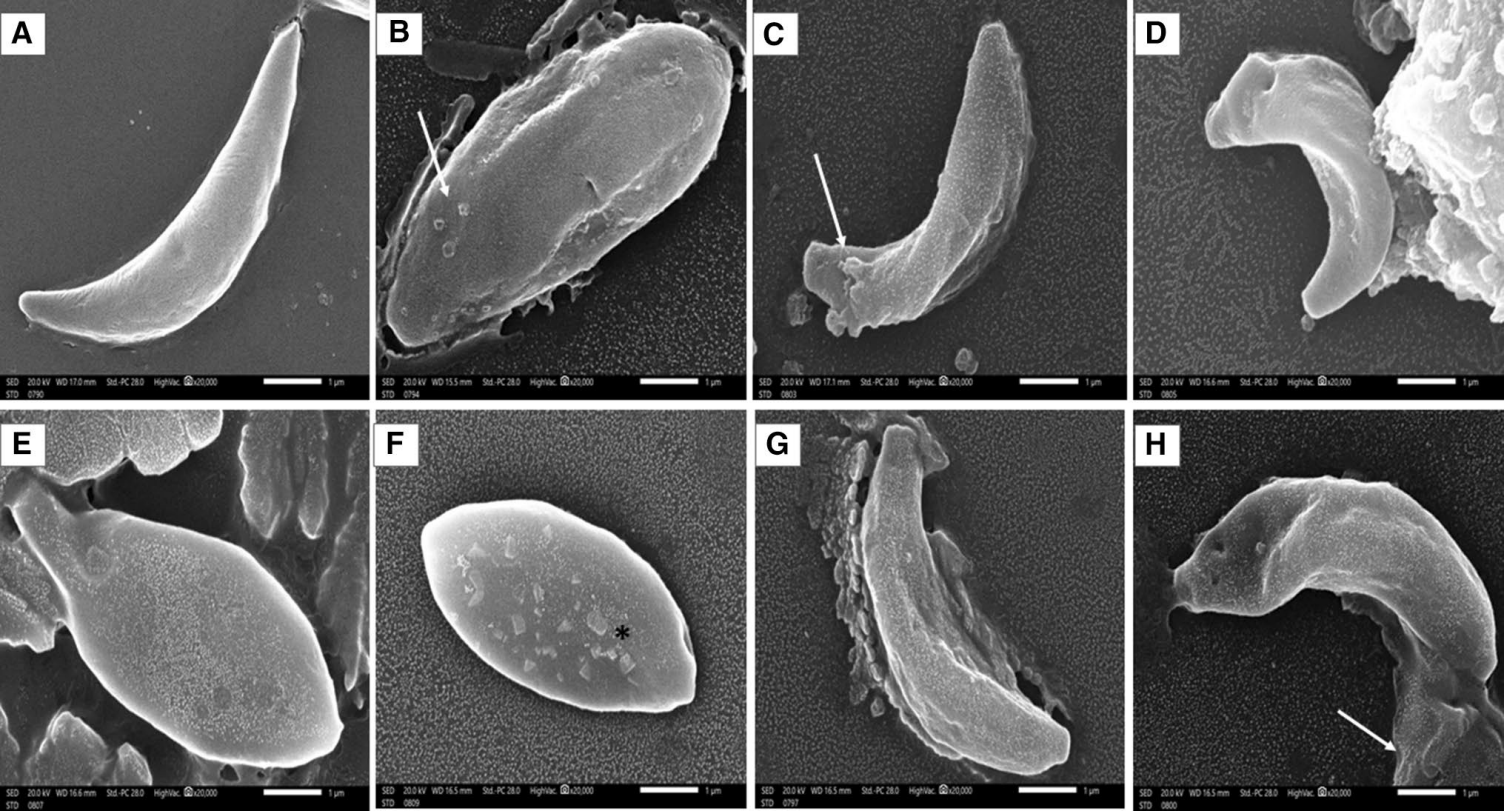
Scanning electron microscopy (SEM) images of *Toxoplasma gondii* tachyzoites showing: **A** non-treated crescent-shaped tachyzoite with smooth regular surface (× 20,000). **B** Tachyzoite treated with sulfadiazine (at 100 µg/ml) showing loss of characteristic crescentic morphology with irregular rough surface, multiple deep ridges and surface blebs (arrow) (× 20,000). **C**, **D** Tachyzoites treated with *Raphanus sativus *(radish) methanol extract (10 µg/m*l*) showing numerous protrusions (arrow), ridges and depressions (× 20,000). **E**, **F** Tachyzoites treated with *Ceratonia siliqua *(carob) methanol extract (10 µg/m*l*) exhibited loss of crescentic morphology and ballooning. Multiple crystalline deposits were noticed on the surface (asterisk) (× 20,000). **G**, **H** Tachyzoites treated with *Cuminum cyminum* (cumin) methanol extract (10 µg/m*l*) revealed irregular rough surface with multiple furrows and deep depressions. Loss of tachyzoite surface integrity with leakage of cytoplasmic contents was also noticed (arrow) (× 20,000)

*Raphanus sativus*, commonly known as radish, is one of the cruciferous vegetables widely consumed all over the world [33]. Earlier literature has recorded several pharmacological effects of *Raphanus sativus,* including antioxidant [34], anti-inflammatory [35], antimicrobial [36], antileishmanial [16], antitumor [37], antiulcer [38] and antiurolithiatic [39] activities. These biological values of radish are attributed to its phytoconstituents of flavonoids, alkaloids, and phenols [40]. The anti-*Toxoplamal* activity of *Raphanus sativus *extract observed in this study may be due to one or more of its bioactive constituents. Phenolic compounds have been demonstrated to exert potent inhibitory effect on *Leishmania donovani* promastigotes and amastigotes by chelating iron, causing morphological alterations and cell cycle disruption [41]. Moreover, prior studies have proved the antagonistic effect of flavonoids against several protozoa such as *Plasmodium falcipar*um*, Entamoeba histolytica, Cryptosporidium parvum*, and *Leishmania donovani* [42–45]. Additionally, it has been announced that alkaloids have a noticeable activity against *Trypanosoma brucei rhodesiense, Leishmania donovani*, and *Plasmodium falciparum* [46–48]. The anti-*toxoplasma* activity of *Raphanus sativus* in this study was ascertained by the ultrastructural alterations noticed by SEM represented as membrane irregularities (protrusions, ridges and depression). These morphological changes may be attributed to its flavonoids content. It has been declared that flavonoids disrupt cytoplasmic and plasma membrane integrity [49, 50]. They also inhibit tyrosine, protein kinases, topoisomerase activity, mitochondrion function, and fatty acid type II synthesis [51, 52] Thereby, they can compromise the mitochondria of tachyzoites and suppress the signaling of cell survival or death factors [53].

*Cuminum cyminum* (Cumin) is an annual aromatic plant belonging to Apiaceae family and possesses diverse nutraceutical and pharmaceutical features [54]. It has been declared to exhibit antioxidant, antibacterial, antifungal, antidiabetic, antistress, hypolipidemic, immunostimulant and memory-improving effects [55, 56]. The main ingredients identified in cumin seed extract were flavonoids, isoflavonoids, flavonoid glycosides, alkaloids, lignins, monoterpenoid glycosides and phenolic compounds [57, 58]. The distinct constituents of cumin may contribute to its anti-*Toxoplasmal* action reported in this study. Nevertheless, further exploration into the mode of action of *Cuminum cyminum* methanol extract against *T. gondii* is needed.

*Ceratonia siliqua* (carob), a member of Leguminosae family, is broadly cultivated in Mediterranean countries, and in some parts of the USA and Australia [59]. Previous reports have revealed antimicrobial, antioxidant, and anticancer effects of carob [60]. Furthermore, *Ceratonia siliqua* has been shown to have potent anthelmintic action against gastrointestinal nematodes (*Haemonchus contortus* and *Trichostrongylus colubriformis*) [61]. The major phenolic components detected in *Ceratonia siliqua* are kaempferol, tannic acid, catechin hydrate and polydatin [62]. The findings of the current research suggested *Ceratonia siliqua* as a promising candidate in combating toxoplasmosis.

Conclusively, the present study elucidated that the methanol extract of *Raphanus sativus*, *Cuminum cyminum* and *Ceratonia siliqua* have significant inhibitory effect on the growth of *T. gondii* RH tachyzoites in vitro. However, future studies are warranted to investigate their efficacy in vivo and to explore their underlying mechanism of action.
